# The Effects of Endophytic *Beauveria bassiana* Inoculation on Infestation Level of *Planococcus ficus*, Growth and Volatile Constituents of Potted Greenhouse Grapevine (*Vitis vinifera* L.)

**DOI:** 10.3390/toxins11020072

**Published:** 2019-01-28

**Authors:** Siphokazi Moloinyane, Felix Nchu

**Affiliations:** Department of Horticultural Sciences, Cape Peninsula University of Technology, Symphony Way, Bellville, P.O. Box 1906, Bellville 7535, South Africa; moloinyanes@gmail.com

**Keywords:** grapevine, volatiles, *Beauveria bassiana*, *Planococcus ficus*, endophytes

## Abstract

Endophytic entomopathogenic fungi are being explored for the management of phytophagous insect pests. The effects of *Beauveria bassiana* (Hypocreales) inoculation of grape plants on the infestation level of *P. ficus*, tissue nutrient contents, and growth and volatile constituents of potted grape plants were assessed. Grapevine plants were individually inoculated with a suspension of 1 × 10^8^ conidia mL^−1^ of *B. bassiana* by drenching before experimentally infesting each of them with thirty adult females of *P. ficus*. At four weeks post-treatment, the fungus was re-isolated from leaves of 50% of the fungus-exposed plants. However, no significant difference (*p* > 0.05) was observed in all the plant growth parameters measured in the fungus-treated and control plants. Plant tissue analysis revealed markedly higher contents of calcium (Ca) and magnesium (Mg) in the leaf tissue of plants exposed to the *B. bassiana* relative to the control. Gas chromatography mass spectrometry (GC-MS) analyses showed that a significantly (*X*^2^ = 5.1; *p* < 0.02) higher number of known anti-insect volatile compounds (nine) were present among fungus treated plants compared to the control plants (five). Naphthalene, which is toxic to insects and humans, was detected only in the volatiles of the fungus-exposed plants. *B. bassiana* did not have any significant effect on total polyphenol, alkaloid, and flavonoids. Overall, treatment with fungus did not inhibit the infestation by *P. ficus*. In conclusion, these findings shed light on some of the mechanisms involved in endophytic fungus-plant-insect interactions.

## 1. Introduction

Numerous setbacks, including pollution, toxicity to animals and plants, and rampant insecticide resistance, are associated with the use of synthetic insecticides [1], and these have led to increased solicitation for alternative solutions to insect infestation problems on crops. Knowledge gained over the years on the influence of endophytic microbial symbionts on plant defense mechanisms vis-à-vis insect herbivory have opened up opportunities for management of insect pests using fungal endophytes. Endophytic fungi occur ubiquitously in plants and colonize them without adverse effects; meanwhile, plants serve as host and provide nutrient to these fungi. Through this mutualistic relationship with their plant hosts, endophytes enhance plants’ tolerance to biotic and abiotic stresses [2]. Endophytic fungi have also been reported to induce increased growth in plants, such as grasses and strawberry [3,4]. The growth enhancing effect can be attributed to the ability of fungi to mobilize valuable nutrients for plant growth; for example, *Metarhizium robertsii* (Hypocreales) promotes root growth and nitrogen absorption in switchgrass and haricot bean [5]. A study by Dara et al. [3] demonstrated improved plant health coupled with an increase in shoot–root ratio for plants treated with the fungus *B. bassiana*. *M. robertsii* and *B. bassiana*, two well-known entomopathogenic fungal species, have strains that can cause natural epizootic deaths in many insects [6]. Plant growth and productivity, as well as defense, could be enhanced by exploring the plant-fungus interaction. 

There are evidences in literature to prove that endophytic fungi can curb insect infestations on plants. For example, western tarnished plant bugs (*Lygus hesperus*) and southern green stink bugs (*Nezara viridula*) showed strong negative responses to flower buds (*L. hesperus*) and fruits (*N. viridula*) from plants that had been colonized by endophytic fungi compared to control plants [7]. A strain of the endophytic fungus *B. bassiana* reduced infestation rates and growth of *P. ficus* on potted grapevine plants [8]. Interesting findings, such as the aforementioned, have spurred researchers to study further the mechanisms through which fungal endophytes influence insect herbivory. Surely, this knowledge will improve our understanding and exploitation of the fungus-plant-insect relationship for sustainable pest management. 

Some endophytic fungi have been reported to produce metabolites that can reduce insect infestations on their host plants [9]. It is believed that increased in quantity and diversity of secondary metabolites in endophyte-containing plants is somewhat responsible for the reduction of insect herbivory on plants [10]. In a study carried out by Jallow et al. [11], *Acremonium strictum* systemically influenced the host selection of *Helicoverpa armigera* moths for oviposition, possibly through changes in volatile emissions and some unknown biochemical parameters. Fungal endophytes can increase the production of antioxidant in plants, as well as produce antioxidant compounds, such as phenolics. They also produce plant growth hormones and enhance plant nutrient absorption, favoring increased germination success and growth rate [12,13]. 

The grapevine mealybug (*Planococcus ficus* [Homoptera]) is a sap-sucking insect that is difficult to control [14,15]; individuals of this species often hide in plant crevices and have a protective waxy layer. It is among the most serious pests of vineyards, causing substantial losses globally [16,17]. The systemic colonization of tissues of young plants with endophytic *B. bassiana* can lead to sustainable control of *P. ficus*. The objectives of this study were to assess the effect of *B. bassiana* inoculation of grape plants on (i) the infestation level of *P. ficus* and (ii) growth and chemical constituents of potted grapevines.

## 2. Results

### 2.1. Re-isolation of Fungus from Grapevine Tissues

At 28 days after inoculation, *B. bassiana* was successfully re-isolated from 29 of 60 leaf sections (48%), representing 10 of the 20 fungus-treated plants (50%). No fungus was re-isolated from the control plants. No microbial contamination was observed in either the control or the treated plants. 

### 2.2. Effect of Fungus on Plant Growth Parameters

There was no significant difference (DF = 1, 38; F = 0.829, *p* = 0.3684) in plant heights between the fungus-exposed plants and control plants at four weeks post-treatment. The heights ranged from 97 ± 9-101 ± 7cm (Table 1). In addition, experimental plants did not show significant variations (*p* > 0.05) between fungus and control treatments for the other plant growth parameters, i.e., leaf number, number of shoots, and wet and dry weights (Table 1).

### 2.3. Effect of Fungus on Plant Tissue Nutrient Content

Generally, fungal inoculation of plants had no significant effect (*p* > 0.05) on the tissue nutrients’ levels. However, calcium (Ca) (16,633 mg/kg) and magnesium (Mg) (3316 mg/kg), were significantly higher in the leaf tissues of the fungus-treated plants (Df = 1, 10; *p* < 0.05) compared to the control plants, which were Ca (13,266 mg/kg) and Mg (2866 mg/kg) (Table 2). 

### 2.4. Effect of the Fungus on the Infestation Level of Grapevine Mealybug

Treatment with fungus did not have any significant (*p* > 0.05) beneficial affect against insect infestations numbers on plants over control; insect infestation numbers for the adult female and larvae ranged from 19 to 18 and 27 to 31, respectively (Table 3). 

### 2.5. Effect on Secondary Metabolite

The fungus had no effect on the total polyphenol content. Alkaloids were not detected (Table 4). A wide range of volatile compounds were detected in the grapevine plants in both treatments (Table 5). Remarkably, a markedly higher (*X*^2^ = 5.1; *p* = 0.02) number of volatile constituents that have been previously reported to have anti-insect activities were detected among fungus treated plants (nine) compared to control plants (five); some of the well-known insect repellents included Limonene, Beta pinene, Naphthalene and gamma-Terpinene (Table 5 and Table 6). 

## 3. Discussion

The re-isolation of the *B. bassiana* from the leaf tissue of 50% of fungus-inoculated plants demonstrated that there was moderate colonization of the tissues of *V. vinifera* plants after experimental inoculation. The colonization of potted grapevine plants by endophytic fungi has been reported previously [9]. It is worth mentioning that successful colonization is influenced by factors, such as fungal species, fungal strain, and host, etc., [28,29,30]. 

Despite successful colonization, interestingly, the colonization by *B. bassiana* of the grapevine tissue did not translate to any noticeable increase in plant growth. Inconsistent effects of fungus on plant growth parameters have been reported. Akello et al. [30] did not observe a major difference in plant growth following inoculation of banana (*Musa* spp.) with endophytic *B. bassiana* strain compared to the control treatment. However, in other similar studies, endophytic *B. bassiana* induced higher growth in onion and tomatoes [31,32]. The growth promoting effects of *B. bassiana* is probably dependent on the availability of nutrients in the growth medium. The absence of visible effects on the growth parameters assessed is seemingly consistent with the no effect of the fungus on the tissue macronutrients (nitrogen (N), phosphorus (P), and potassium (K)) contents observed in this study—plant growths are known to be correlated with these primary nutrients [33]. Fungal microbes are generally known to promote plant growth by facilitating nutrient resource acquisition from the environment. In the current study, the plants were exposed to a constant and adequate supply of the required nutrients, especially nitrogen, and this might have reduced any influence the fungus might have had on the supply of nutrients to plants. According to Tall and Meyling [34], the mechanisms by which *B. bassiana* increases plant growth should be investigated further. 

The two nutrients that were found to be significantly influenced by fungal inoculation were calcium and magnesium. These nutrients are important to plants for varying reasons. Calcium is an essential element for the development of new plant tissues; it strengthens cell walls and promotes cell elongation [35]. It is also known to help protect plants against pathogenic fungi and bacteria. Magnesium is a building block for chlorophyll, which is important for the process of photosynthesis. It also activates certain plant enzymes needed for growth and contributes to protein synthesis [36]. They can also influence secondary metabolite production. For example, supplementing plants with Ca has been proven to enhance total protein content, as well as phenol and flavonoid content [37]. Although little is known about the role of Mg in plant secondary metabolism, Mg deficiency is known to increase phenolic compounds and putrescine accumulation in cells [38].

The key finding here is the clear association between the number of volatiles detected and the fungus treatment (Table 5). There are many plausible explanations for the higher volatiles in the fungus-treated plants: perhaps this is mediated through calcium and magnesium uptake [37,38], maybe it is due to direct production of secondary metabolites by fungi [39], or better still, it is as a result of plant defense reaction in the presence of the fungus in the tissue [40]. Interestingly, naphthalene was detected in the fungus treated plants in this study. Naphthalene is toxic to humans, is a potent insecticide and an insect repellent [41,42], and is the main active ingredient in mothballs. In a separate study, *B. bassiana* released volatile organic compounds, including diisopropyl naphthalenes [43]. Earlier, Daisy et al. [24] demonstrated that *Muscodor vitigenus*, an endophytic fungus, can also produce naphthalene and further showed that the insect repellent activities of the fungus against adult stage of the wheat stem sawfly, *Cephus cinctus*, was comparable to authentic naphthalene. 

Plants have evolved several responses and defenses against biotic and abiotic stresses; the production of volatiles is an important and immediate response. These volatiles are known to be involved in communications with natural enemies of particular insect herbivores; they have the potential to enhance the effectiveness of host plant resistance to herbivory. While some of these volatiles are known to possess anti-insect properties (Table 6), for examples, naphthalene and limonene, have been reported to cause up to 90–100% insect mortalities in *Tribollium castaneum* Herbst and mealybugs, respectively [41,44], no protective effect against *P. ficus* infestation (fungus treatment; immature (19 ± 1.3), adult (27 ± 4), and control treatment; immature (31 ± 5) and adult (18 ± 2.2)) was observed in this study, despite the higher variety of anti-insect volatile compounds in the fungus treatment. It is worth remembering that insects have co-evolved mechanisms to overcome plant defense mechanisms [45].

## 4. Conclusions

In conclusion, this is the first report of the direct influence of *B. bassiana* on grapevine production of anti-insect volatile compounds and the tissue nutrient contents of grapevine plants. These findings contributed to our understanding of the mechanisms involved in the fungus–plant–insect relationship, as well as highlight the potential risks of mycotoxin contamination in plants that are exposed to fungal endophytes.

## 5. Materials and Methods

### 5.1. Plant Material

Fifty two-year-old *Vitis vinifera* L. cv Pinotage grafted onto Ramsey rootstock plants were obtained from Bosman Family vineyards, Wellington, South Africa. These plants were transported to the greenhouse at Cape Peninsula University of Technology (CPUT), Bellville, South Africa. The plants were debagged and the soil washed off the roots of each plant. All plants were measured to obtained baseline plant height, root length, leaf number, and shoot number prior to their use in the green house experiment. Ten of these plants were used to obtain baseline data (dry weight and wet weight) and the remaining 40 plants were used for the greenhouse experiment.

### 5.2. Fungal Culture

An indigenous *B. bassiana* (strain: SM3) that was originally isolated from a soil sample collected from a vineyard in the Cape Winelands and was found to be pathogenic against the grapevine mealybug (*P. ficus*) was used in this study [46]. This strain was identified using molecular (GenBank accession no. MH598822; Btub and MH595805; ITS) and morphological techniques described in White et al. [47], Glass and Donaldson [48] and Abaajeh and Nchu [49]. Briefly, the fungal morphology was determined using a Nikon Eclipse E800 light microscope. DNA was extracted from the fresh cells, checked on a 1% agarose gel, stained with ethidium bromide, and the gene regions internal transcribed spacer (ITS) (ITS1 (forward) ITS4 (reverse)), the b-tubulin gene (Bt2a (forward), and Bt2b (reverse)) were used for comparison. The sequences were compared using BLAST on the NCBI GenBank database. The DNA sequences of the fungal isolate were submitted to NCBI GenBank and the accession numbers obtained. Cultures of the fungus are being maintained at Cape Peninsula University of Technology in Bellville, South. Clean single-conidia sub-cultures of the fungus were cultivated on half-strength Potato Dextrose Agar (PDA) containing 0.02 g/L of ampicillin (Sigma-Aldrich) and 0.04 g/L streptomycin (Sigma-Aldrich, Johannesburg, South Africa) in 9 and 14 cm diameters Petri dishes and incubated in the laboratory at 25 °C and 12:12 h L:D. Four weeks (28 days) old *B. bassiana* conidia obtained from PDA plates were used for inoculation of the grapevine plants. Conidia were harvested by gently scraping the surface of the Agar using a spatula onto sterile aluminium foil. The conidia were suspended in 500 mL glass bottles containing sterile 0.01% Tween 80 in distilled water. Bottles were capped, and mixed by agitating for five min by shaking and using a magnetic stirrer (at 20 °C and 300 rpm for 30 min) to homogenize the conidial suspension. The conidia concentration was enumerated using a haemocytometer and observed with a light microscope at 40× magnification. In order to obtain the desired concentration (1 × 10^8^ conidia mL^−1^), the volume of sterile 0.01% Tween 80 was increased or conidia were added to the glass bottle. A conidial germination test to determine conidial viability was carried out, according to the method described by Inglis et al. [50], and high spore germination of more than 90% was obtained.

### 5.3. Greenhouse Experiment

This experiment was conducted at the greenhouse of the Department of Horticultural Sciences, Cape Peninsula University of Technology (CPUT), South Africa. The experiment was carried out under the following conditions: An average day temperature of 25 ± 5 °C and average RH of 65 ± 5% between March and April 2018. *V. vinifera* plants were transplanted separately into 40 glass bottles containing a plant growth medium consisting of a mixture of inert substrate materials, vermiculite and perlite in a ratio of 1:1. The bottles were covered with black cloth to prevent algal growth. Twenty potted plants were drenched with 200 mL suspension of *B. bassiana* at 1 × 10^8^ conidia mL^−1^. Twenty control plants were drenched with the 200 mL of sterile distilled water containing 0.01% Tween 80 only. Throughout the experiment, the plants were fertigated weekly with a hydroponic fertilizer, Nutrifeed (Starke Ayres, Cape Town, South Africa), containing 65 g/kg N, 27 g/kg P, 130 g/kg K, 70 mg/kg Ca, 20 mg/kg Cu, 1500 mg/kg Fe, 10 mg/kg Mo, 22 mg/kg Mg, 240 mg/kg Mn, 75 mg/kg S, 240 mg/kg B, and mg/kg Zn. The nutrient solution was prepared by dissolving 60 g of the fertilizer in 60 L reservoir with tap water, and each plant was hand-fed with 500 mL every week. After two weeks, all experimental plants were infested with female adult mealybugs (30 per plant) by transferring insects onto plants using a soft brush [51]. Plants in test and control treatments were inoculated for a second time by drenching with a suspension of 200 mL *B. bassiana* at 1 × 10^8^ conidial mL^−1^ in 0.01% Tween 80 and 0.01% Tween, respectively. On the third week, fungal colonization was assessed as described below (see Section 5.4). The experiment ran for four weeks, after which plant growth parameters (shoot height, number leaves, number of shoots, root length, and shoot and root fresh and dry weights) were recorded. Only plants that showed evidence of successful fungal colonization were selected to represent the fungus treatment thereafter. Mealybug infestation levels were assessed by counting numbers of adult females and larvae on control and fungus-exposed plants. Six representative potted live plants; i.e., three from each treatment, were taken to the central analytical facilities, GC-MS Unit, Stellenbosch University for GC-MS analysis. Fresh leaf samples representing six control samples and six test samples were sent to Bemlab, a commercial testing laboratory in Somerset West, Western Cape, for tissue analysis of nutrient content. The remaining plants were oven-dried separately, at 25 °C for seven days, and then ground with a Jankel and Kunkel Model A 10 mill into fine powder. One hundred milligrams (100 mg) of powdered material from each of 10 samples in control and 10 in the test groups was analyzed for secondary metabolites (total alkaloid, polyphenol, and flavonol) at the Oxidative Stress Centre at CPUT. 

### 5.4. Re-Isolation of B. Bassiana

Endophytic colonization of *B. bassiana* of leaf was assessed at 21 days by re-isolation following surface sterilization. One leaf was carefully excised from each plant and transferred to the laboratory on ice. From each leaf, rectangular leaf sections of 2 mm^2^ were cut. These sections were individually surface-sterilized with 0.5% sodium hypochlorite for 1 min, followed by 70% Ethanol for 1 min and rinsed twice in sterile distilled water and placed on the selective medium (19.5 g Potato Dextrose Agar [PDA], 0.02 g/L of ampicillin (Sigma-Aldrich), and 0.04 g/L streptomycin [Sigma-Aldrich, Johannesburg, South Africa]). The leaf sections were examined visually on a daily basis for presence of any fungal growth. Fungal tissue was characterized as endophytic by observing *B. bassiana* white dense mycelia becoming creamy at the edge growing from the tissues of sterilized leaf sections. A total of 40 (20 control and 20 treatment) plants were sampled and 120 leaf sections were plated, equating to three leaf sections per plant. The presence of *B. bassiana* in at least one of the leaf sections was considered an indication of successful colonization of a plant. The data was expressed as percentage colonization (number of plant replicates colonized/number of plant replicates excised ×100).

### 5.5. Plant Growth

The effect of *B. bassiana* on the growth of *V. vinifera* L. was determined by measuring shoot height, number leaves, number of shoots, root length, and shoot and root fresh and dry weights. Plant height was measured from the surface of the substrate to the tip of the stem.

### 5.6. Plant Tissue Nutrient Analysis

Leaf samples were analyzed for macro and micro at Bemlab (Pty) Ltd. (Somerset West, Cape Town, South Africa), a commercial laboratory. Leaves were washed with Teepol solution, rinsed with de-ionised water, and dried at 70 °C overnight in an oven. The dried leaves were then milled and ashed at 480 °C, and shaken up in a 50:50 HCl (50%) solution for extraction through filter paper [52,53]. The potassium (K), phosphorus (P), Ca, Mg, sodium (Na), manganese (Mn), iron (Fe), copper (Cu), zinc (Z), and boron (B) contents of the extracts were analyzed using the Ash method. The total nitrogen (N) content of the leaves was determined through total combustion in a Leco N-analyser. The amounts of N, P, K, Ca, and Mg were converted from percentage (%) to mg/kg. 10,000 was used as the conversion factor.

### 5.7. Analysis of Secondary Metabolites

Six representative potted live plants, i.e., three from each treatment, were used for this analysis. Only plants that showed evidence of successful fungal colonization were selected to represent the fungus treatment in the GC-MS analysis.

#### 5.7.1. GC-MS Analysis (Headspace) and Secondary Metabolite Analysis

##### Sample Preparation

Whole leaves were cut-off from fresh plants and freeze-dried overnight at −80 ˚C. The leaves were then crushed using liquid nitrogen, and 1 g was weighed into a solid phase micro-extraction (SPME) vial followed by 2 mL of 12% alcohol solution (*v*/*v*) at pH 3.5 and 3 mL of 20% NaCl solution. The samples were vortexed and the headspace of the sample was analyzed using a Divinylbenzene/Carboxen/Polydimethylsiloxane (DVB/CAR/PDMS) SPME fiber (gray).

#### 5.7.2. Chromatographic Separation

Separation of volatile compounds was performed with a gas chromatograph (6890N, Agilent Technologies Network) coupled to an Agilent Technologies Inert XL EI/CI Mass Selective Detector (5975B, Agilent Technologies Inc., Palo Alto, CA, USA). The GC-MS system was coupled to a CTC Analytics PAL autosampler, and the separation of volatiles present in the samples was achieved on a polar ZB-WAX (30 m, 0.25 mm ID, 0.25 µm film thickness) Zebron 7HG-G007-11 capillary column. Helium was used as the carrier gas at a flow rate of 1 mL/min. The injector temperature was kept at 250 °C with a split ratio of 5:1. The oven temperature was programmed as follows: 35 °C for 6 min, at a rate of 3 °C/min to 70 °C for 5 min, then at 4 °C/min to 120 °C for 1 min, and finally ramped up to 240 °C at a rate of 20 °C/min and maintained for 2.89 min. The Mass Selective Detector was operated in a full scan mode, and the source and quad temperatures were maintained at 230 °C and 150 °C, respectively. The transfer line temperature was maintained at 250 °C. The mass spectrometer was operated under electron impact mode at ionization energy of 70 eV, scanning from 35 to 500 *m*/*z*. Relative ratios were calculated using the expression (peak area/IS peak area) × IS concentration (IS = internal standard), and hence are only approximate values. Only the organic volatile compounds with a match quality of at least 90% were identified and reported.

#### 5.7.3. Determination of Total Flavonol, Alkaloid, and Phenolic Content

A spectroscopic method was used to determine total alkaloids in the plant [54]. Briefly, 100 mg of the powdered grapevine leaves were extracted with 10 mL of 60% ethanol for 2 h, centrifuged (4000× *g* for 10 min), and the supernatant was used in the assay. Thereafter, 2 mL of the extract supernatant and atropine standard solutions were mixed with 5 mL sodium phosphate buffer and 12 mL bromocresol green solution. Twelve milliliters of chloroform were then added to the solution, and the solution was mixed vigorously using a vortex mixer. The absorbance at 417 nm was determined, and the concentration of the sample (mg/g) using a standard curve of atropine was calculated. The total polyphenol content of the various crude extracts was determined by the Folin–Ciocalteu method [55]. Using a 96-well microplate, 25 μL of the sample was mixed with 125 μL Folin–Ciocalteu reagent (diluted 1:10 with distilled water) (Merck, Cape Town, South Africa). After 5 min, 100 μL (7.5%) aqueous sodium carbonate (Na_2_CO_3_) (Sigma-Aldrich, South Africa) was added to each well. The method described by Daniels et al. [56] was used to obtain the absorbance reading of the solution in the microplates, and results were expressed as mg gallic acid equivalents per g dry weight (mg GAE/g DW). The flavonol content was determined using quercetin, and the protocol was based on the method described by Daniels et al. [57]. In the sample wells, 12.5 μL of the crude sample extracts was mixed with 12.5 μL 0.1% hydrochloric acid (HCl) (Merck, Cape Town, South Africa) in 95% ethanol and incubated for 30 min at room temperature. The results were expressed as mg quercetin equivalent per g dry weight (mg QE/g DW).

### 5.8. Statistical Analysis

The experimental data collected were analyzed using one-way analysis of variance (ANOVA) and Tukey HSD test was used to separate the means at a level of significance, *p* < 0.05. These computations were performed using PAST version 3.21 (Øyvind Hammer, Oslo, Norway) [58]. The Pearson Chi-square test was used to compare the number of volatiles produced by plants in fungus and control treatments.

## Figures and Tables

**Table 1:** Mean growth ±SE of *V. vinifera* exposed to *B. bassiana* inoculum and control treatment for four weeks under greenhouse conditions.

Treatment	Plant Height (cm)	Leaf Count	Number of Shoots	Dry Weight Roots (g)	Dry Weight Shoots (g)	Wet Weight Roots (g)	Wet Weight Shoots (g)
Fungus	97 ± 9a	30 ± 3a	3 ± 0.2a	8 ± 0.6a	66 ± 49a	35 ± 3a	31 ± 3a
Control	101 ± 7a	25 ± 4a	3 ± 0.3a	8 ± 0.9a	17 ± 2a	33 ± 2a	33 ± 4a

a: Means followed by same lowercase letter in a column (Table 1) are not significantly different (*p* > 0.05) following comparison of fungus and control treatments using Tukey’s test.

**Table 2:** Tissue nutrients contents (Mean ± SE) in shoots of *V. vinifera* plants exposed to control and *B. bassiana* inoculum for four weeks under greenhouse conditions. Nitrogen = N, phosphorus = P, potassium = K, calcium = Ca, magnesium = Mg, sodium = Na, manganese = Mn, iron = Fe, copper = Cu, zinc = Z, and boron = B.

Treatment	Quantity (mg/kg)										
	N	P	K	Ca	Mg	Na	Mn	Fe	Cu	Zn	B
Control	23,433 ± 912a	5900 ± 717a	27,583 ± 1200a	13,266 ± 939b	2866 ± 158b	6624 ± 337a	47 ± 4a	321 ± 65a	4 ± 0.2a	60 ± 3a	26 ± 1a
Fungus	23,500 ± 657a	4500 ± 265a	27,450 ± 987a	16,633 ± 544a	3316 ± 47a	8446 ± 760a	49 ± 3a	243 ± 13a	5 ± 0.1a	68 ± 2a	27 ± 0.5a

a,b: Means followed by same lowercase letter in a column (Table 2) are not significantly different (*p* > 0.05) following comparison of fungus and control treatments using Tukey’s test.

**Table 3:** Mean number of *Planococcus ficus* (immature [larvae] and adult females) on the control and the *Beauveria bassiana*-inoculated plants at four weeks after the commencement of experiment under greenhouse conditions (6 replicates per treatment).

Treatment	Mean No. of Insects	
	Immature	Adult
Fungus	27 ± 4	19 ± 1.3
Control	31 ± 5	18 ± 2.2

**Table 4:** Secondary metabolite contents in shoots of grapevine following exposure to *B. bassiana* inoculum and control treatment.

Treatment	Polyphenol (mg GAE/g)	Flavonols (mg QE/g)	Alkaloids (mg AE/g)
Control	8 ± 0.5	3 ± 0.3	N.D.
Fungus	8 ± 0.5	3 ± 0.3	N.D.

N.D.: Not detected.

**Table 5:** Volatile organic compounds with a match quality of at least 90% present in fungal treatment and control shoots of grapevine.

Control	Fungus
1,2–Benzenedicarboxylic acid *	1–Hexanol
1–Hexanol	2–Heptenal
1–Octadecene *	2–Hexen–1–ol
2–Furancarboxaldehyde *	2–Hexenoic acid *
2–Heptenal	3–Hexen–1–ol
2–Hexen–1–ol	3–Hexenoic acid
3–Hexen–1–ol	6–Methyl–5–Hepten–2–one
3–Hexenoic acid	alpha.–Terpinolene *
6–Methyl–5–Hepten–2–one	Benzaldehyde
Benzaldehyde	Benzene *
Benzeneethanol	Benzeneethanol
Benzoic acid *	Benzofuranone *
Benzyl alcohol *	beta–Pinene
beta–Pinene	Butanoic acid *
CIS–3–Hexenol	CIS–3–Hexenol
CIS–3–Hexenyl Caproate *	CIS–3–Hexenyl alpha. Methyl butyrate *
CIS–3–Hexenyl ISO–Butyrate *	CIS–3–hexenyl Valerate *
Citral	Citral
Cyclododecane *	Cyclohexasiloxane
Cyclohexadecane *	Cyclooctatetraene *
Cyclohexasiloxane	Cyclopentasiloxane *
Decanal *	Cyclotetrasiloxane *
Dodecanoic acid	delta–cadinene *
Farnesene *	E–3–hexenyl hexanoate *
gamma–Bisabolene *	Ethyl phthalate *
gamma–Terpinene	Ethylidenecyclohexane *
Geraniol	Farnesyl acetone *
Geranylacetone	gamma–Terpinene
Heptadecanoic acid *	Geranial *
Hexadecanoic acid *	Geraniol
Hexanal	Geranylacetone
Limonene	Heptadecene *
Linoleic acid *	Hexanal
Muskolactone *	Limonene
Myrcene	m–Cymene *
Myristic acid *	Myrcene
Octadecanoic acid *	Naphthalene *
Octanal	Nerolidol *
Oleic acid *	Octanal
p–Cymene *	Pentanoic acid *
Pentadecanoic acid *	Phenylethyl alcohol*
Pentenal *	Styrene
Squalene *	Tetradecamethylcycloheptasiloxane
Styrene	Trans 2–Hexenoic acid *
Tetradecamethylcycloheptasiloxane	Trans,Trans–2,4–Heptadienal *
Thiosulfuric acid *	Trans–beta–Ocimene *
Trans–2–Hexenal *	
Trans–Geraniol *	
Z–3–hexenyl 2–methylbutanoate *	

*: A compound in Table 5 that was only detected in either the control or fungus treated plant(s).

**Table 6:** Selected well-known and published anti-insect volatiles that were also detected in *Vitis vinifera* in this study and their relative area ratios following gas chromatography-linked mass spectrometry analysis of control and fungus-treated plants.

Anti-Insect Compound	Reference	Area Ratio Control	Area Ratio Fungus
Benzaldehyde	Paulraj et al. [18]	0.23 ± 0.1a	0.24 ± 0.04a
Limonene	Hebeish et al. [19]	4.5 ± 1.2a	2.4 ± 1.3a
Geraniol	Maia and Moore [20] Chen and Viljoen [21]	0.19 ± 0.04a	0.29 ± 0.05a
Geranylacetone	Maia and Moore [20]	-	0.52 ± 0.1a
gamma-Terpinene	Wang et al. [22]	-	1.48 ± 0.4a
beta-Pinene	Dambolena et al. [23]	0.94 ± 0.21a	0.41 ± 0.25a
Napthelene	Daisy et al. [24]	-	0.23 ± 0.02a
M-Cymene	Chang et al. [25]	-	0.5 ± 0.1a
Citral	Oyedele et al [26]; Abdel-Tawab and Mossa [27]	0.03 ± 0.03a	0.06 ± 0.06a
No. of compounds		5	9 *

* Denotes significantly higher (DF = 1; *X*^2^ = 5.1 and *p* = 0.02) number of compounds present following Pearson Chi square test in Table 6. a: Means followed by same lowercase letter in a column (Table 6) are not significantly different (*p* > 0.05) following comparison of fungus and control treatments using Tukey’s test.
